# German Version of the Mobile Agnew Relationship Measure: Translation and Validation Study

**DOI:** 10.2196/43368

**Published:** 2023-11-13

**Authors:** Clemens von Wulffen, Marta Anna Marciniak, Judith Rohde, Raffael Kalisch, Harald Binder, Oliver Tuescher, Birgit Kleim

**Affiliations:** 1 Department of Psychology University of Zurich Zürich Switzerland; 2 Department of Psychiatry, Psychotherapy and Psychosomatics Psychiatric University Hospital University of Zurich Zurich Switzerland; 3 Leibniz Institute for Resilience Research Mainz Germany; 4 Neuroimaging Center Focus Program Translational Neuroscience Johannes Gutenberg University Medical Center Mainz Germany; 5 Institute of Medical Biometry and Statistics Faculty of Medicine and Medical Center University of Freiburg Freiburg Germany; 6 Freiburg Center for Data Analysis and Modelling University of Freiburg Freiburg Germany; 7 Department of Psychiatry and Psychotherapy University Medical Center University Johannes Gutenberg University Mainz Germany

**Keywords:** therapeutic alliance, digital therapeutic alliance, mental health apps, mHealth, mobile health, translation, validation, mobile phone

## Abstract

**Background:**

The mobile Agnew Relationship Measure (mARM) is a self-report questionnaire for the evaluation of digital mental health interventions and their interactions with users. With the global increase in digital mental health intervention research, translated measures are required to conduct research with local populations.

**Objective:**

The aim of this study was to translate and validate the original English version of the mARM into a German version (mARM-G).

**Methods:**

A total of 2 native German speakers who spoke English as their second language conducted forward translation of the original items. This version was then back translated by 2 native German speakers with a fluent knowledge of English. An independent bilingual reviewer then compared these drafts and created a final German version. The mARM-G was validated by 15 experts in the field of mobile app development and 15 nonexperts for content validity and face validity; 144 participants were recruited to conduct reliability testing as well as confirmatory factor analysis.

**Results:**

The content validity index of the mARM-G was 0.90 (expert ratings) and 0.79 (nonexperts). The face validity index was 0.89 (experts) and 0.86 (nonexperts). Internal consistency for the entire scale was Cronbach α=.91. Confirmatory factor analysis results were as follows: the chi-square statistic to df ratio was 1.66. Comparative Fit Index was 0.87 and the Tucker-Lewis Index was 0.86. The root mean square error of approximation was 0.07.

**Conclusions:**

The mARM-G is a valid and reliable tool that can be used for future studies in German-speaking countries.

## Introduction

According to the World Health Organization, mental health conditions are increasing globally, with an estimated 20% of children and adolescents having a mental health condition and suicide being the second-leading cause of death among those aged 15-29 years [1]. In order to address this increasing need, the digitalization of psychotherapeutic interventions and mental health-related monitoring holds enormous potential to be implemented either in addition to clinical interventions or as a substitute altogether. Unlike traditional therapeutic methods, digital implementations may be realized on a much larger scale as they are not restricted by potentially limiting factors such as time constraints, availability of therapists or clinicians, monetary considerations, or fear of stigmatization. In addition, mobile mental health interventions can be applied in everyday situations [2].

Yet, despite the increase in commercial app development in the field of mental health, which has resulted in currently more than 10,000 mental health apps available in the market [3], there has been a striking lack of empirical studies investigating the effectiveness of these novel apps [4], leading to serious concern about their scientific credibility. More research is therefore required to evaluate the effectiveness and suitability of these mental health apps. One of the key aims in this context has been to identify factors contributing to the potential success and effectiveness of mental health apps.

Therapeutic alliance (TA) refers to the working relationship between client and therapist [5]. In face-to-face therapeutic settings, TA has been suggested to be an important contributor to therapeutic success [6]. For example, a recent systematic review by Baier et al [7] found TA to mediate positive outcomes by over 70%. Moreover, a longitudinal study reported patients’ and therapists’ positive perception of TA to predict clinical improvement in patients’ depressive outcomes [8], and another study reported TA to have a strong association with youth mental health and addiction treatment outcomes [9], which has also been found for drug abuse treatment altogether [10].

TA has been increasingly researched for digital therapy with video therapy [11,12] and text-based therapy [13]; however, questions still pertain as to whether it is possible to build a relationship between a user and an app in the context of a mobile health (mHealth) intervention, and if so, what would be the nature of such a rapport? The findings have so far been mixed. Some research found associations between digital TA and treatment outcomes using fully automated smartphone apps [14] while other studies reported a lack of such association [15]. As such, there is currently no consensus regarding the nature and potential impact of digital TA on treatment outcomes [16].

One of the limiting factors that has prevented adequate investigations in this regard has been the lack of suitable measures to assess digital TA [16]. Some authors used measures from traditional face-to-face therapy [15], whereas others used altered versions of these instruments [17]. However, several reviews noted the need to substantially revise and adopt new measures to adequately measure the nature of TA for app-based interventions [16,18]. To address this, the Agnew Relationship Measure (ARM)—a common tool used to assess TA in traditional face-to-face settings [19]—has been modified into the mobile Agnew Relationship Measure (mARM) to measure TA in digital settings [20]. The mARM consists of 25 items that are rated on a 7-point Likert scale, ranging from (1) strongly disagree to (7) strongly agree. Like the ARM, the mARM is built upon five key concepts of TA, which include (1) the bond between the app and user; (2) the partnership or collaboration between user and app; (3) the confidence the user holds in the competency of the app; (4) the openness or freedom the user feels to personally disclose; and (5) the client or user initiative, which refers to feelings of control and empowerment within the relationship.

The mARM has been discussed in several reviews regarding digital innovation in psychological practice [18,21-24], as well as being the focal point of a recent paper on human-computer interaction [25].

To date, the mARM is only available in English, and the aim of this study was to translate the mARM into German and consequently validate the new version so that it can be used for research in German-speaking countries.

## Methods

### Translation and Adaptation Process

**Figure 1:**
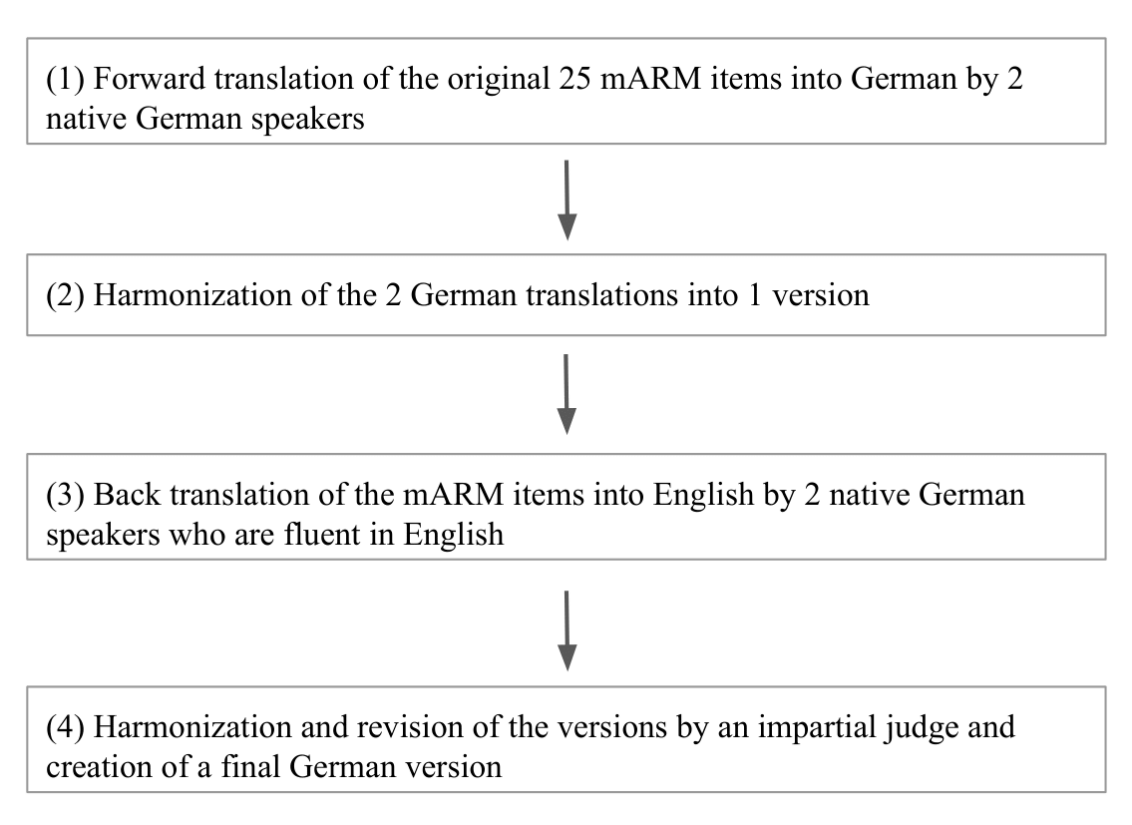
The translation process of the mARM into the mARM-G. mARM: mobile Agnew Relationship Measure; mARM-G: mobile Agnew Relationship Measure German version.

The translation process was modeled after the guidelines by Beaton et al [26]. After having obtained permission to translate the mARM from its first author, the items of the mARM were forward translated from English into German separately by 2 researchers who are native German speakers and who speak English as their second language. These 2 versions of the German translation were then compared and harmonized by another researcher. The resulting version was then back translated into English by 2 native German speakers with a fluent knowledge of English. Following this, an impartial judge who is bilingual in German and English reviewed the versions and created a final mARM German version (mARM-G; Figure 1).

### Validation and Reliability Testing and Data Analysis

The mARM-G underwent a validation process that included testing for content validity, face validity, and reliability. Content validity aims to measure the relevancy of each item for TA. This was assessed according to the content validity index (CVI) [27]. The relevancy of each item of the mARM-G was measured with regard to the TA, conducted on a 4-point Likert scale ranging from 1 (not relevant) to 4 (very relevant). First, average ratings and SDs for all answers were computed to provide a general overview. Then, in order to calculate the CVI, all scores of 3 to 4 were categorized as relevant (1), and all scores of 1 and 2 were categorized as not relevant (0). The CVI was calculated for each item (item-CVI) and for the entire scale by averaging the scores of all items.

Face validity aims to measure the comprehensibility and clarity of each translated item of the mARM-G. This was done using a 4-point Likert scale ranging from 1 (not clear) to 4 (very clear). Average ratings for all answers as well as SDs were calculated. The face validity index (FVI) was then computed by coding all scores of 3 to 4 as clear (1), and all scores of 1 and 2 as not clear (0). The FVI was then calculated for each item (item-FVI) and for the entire scale by averaging the scores of all items.

Reliability testing was performed by measuring the internal consistency of the items of the mARM-G via Cronbach α. Sample size was estimated according to the method provided by Bonett [28] with the use of a web-based sample size calculator [29]. The Cronbach α was set to .7 with a precision of 0.1 and a 95% CI. The minimal required sample size was 77 participants. Finally, confirmatory factor analysis was conducted using the *lavaan* package in R (Ghent University) [30] and visualized using the *semPlot* function [31]. For this, the sample size was estimated for root mean square error of approximation (RMSEA) based on the resources by Preacher and Coffman [32]. Parameters set with α level of .05, df of 265, a null hypothesized RMSEA value of 0.05, and an alternative RMSEA value of 0.07. Based on this, a sample size of 130 participants was required to reach a power of 0.80. All analyses were performed using R in RStudio (version 4.1.1; R Core Team) [33].

### Participants

The 15 experts who conducted the content and face validation were all native German speakers living in Germany and the German-speaking part of Switzerland. The group consisted of clinical psychologists (n=7, 47%) who conduct psychological research as well as scientists (n=8, 53%) specializing in psychology research. This included 6 (40%) psychologists at the doctoral level, 5 (33%) at the postdoctoral level, and 4 (27%) full professors. A total of 11 (73%) experts were female and 4 (27%) were male, with a mean age of 35 (SD 9.2) years.

The 15 nonexperts who conducted content and face validation were all native German speakers living in Germany (n=11, 73%), the German-speaking part of Switzerland (n=3, 20%), and the United Kingdom (n=1, 7%). A total of 5 (33%) were female, 2 (13%) preferred not to reveal their gender, and 8 (53%) were male. The mean age was 27.53 (SD 9.7) years.

During the study, participants were using the mental health app—ReApp [34]. ReApp is based on principles of cognitive behavioral therapy and reappraisal. Full development protocol can be found elsewhere [35]. The participants used the app for 21 days before completing the mARM-G.

### Ethical Considerations

The reliability analysis was based on 144 participants. The Ethics Committee for the Faculty of Arts and Social Sciences of the University of Zurich approved the study proposal (21.2.12). All participants were students and spoke fluent German, and the exclusion criteria were having a mental illness as reported by the participant and attending psychotherapy or using other kinds of support from a qualified psychologist or psychiatrist. All participants provided written informed consent to participate in the study and agreed to data analysis by researchers from the University of Zurich. To ensure the privacy of the participants, they were provided with their own personal code to use throughout the study. Participants were remunerated up to 105 Swiss francs (around US $114) or 6 university credit points to compensate for their contribution to the study.

## Results

For the expert group, the overall average of the relevancy rating was 3.52 (SD 0.29; range 1-4). The CVI average for the scale was 0.9 (SD 0.12). The overall average of face validity or clarity ratings was 3.6 (SD 0.35; range 1-4). The FVI average for the entire scale was 0.89 (SD 0.15). For all ratings, see Table 1. All items with scores below 0.78 for the FVI and CVI were further inspected, and adjustments were made to make the translated items clearer and more relevant (Multimedia Appendices 1 and 2).

**Table 1:** Experts (n=15) rating for content validity and face validity.

Items	Relevancy of each item, mean (SD)	I-CVI^a^	Clarity of each item, mean (SD)	I-FVI^b^
1	3.40 (0.74)	0.93	3.13 (0.99)	0.73
2	3.53 (0.83)	0.93	3.73 (0.59)	0.93
3	2.80 (0.77)	0.6	2.33 (0.90)	0.33
4	3.73 (0.59)	0.93	3.67 (0.62)	0.93
5	3.87 (0.52)	0.93	3.87 (0.52)	0.93
6	3.27 (0.46)	1	3.53 (0.74)	0.86
7	3.87 (0.35)	1	3.73 (0.46)	1
8	3.87 (0.35)	1	3.93 (0.26)	1
9	3.93 (0.26)	1	3.87 (0.35)	1
10	3.67 (0.49)	1	3.80 (0.41)	1
11	3.60 (0.83)	0.93	3.73 (0.46)	1
12	3.20 (0.86)	0.73	3.07 (1.03)	0.66
13	3.73 (0.46)	1	3.87 (0.35)	1
14	3.60 (0.63)	0.93	3.73 (0.70)	0.86
15	3.13 (0.92)	0.66	3.53 (0.74)	0.86
16	3.80 (0.41)	1	3.87 (0.35)	1
17	3.60 (0.63)	0.93	3.80 (0.56)	0.93
18	3.60 (0.63)	0.93	3.53 (0.74)	0.86
19	3.53 (0.52)	1	3.73 (0.46)	1
20	3.33 (0.72)	0.86	3.67 (0.62)	0.93
21	3.00 (0.76)	0.73	3.87 (0.35)	1
22	3.67 (0.49)	1	3.40 (0.83)	0.8
23	3.53 (0.64)	0.93	3.47 (0.74)	0.93
24	3.33 (0.82)	0.8	3.40 (0.99)	0.8
25	3.33 (1.05)	0.733	3.73 (0.59)	0.93
Measure average	3.52	0.9	3.60	0.89

^a^I-CVI: item–content validity index.

^b^I-FVI: item–face validity index.

For the nonexperts group, the overall average of the item relevancy rating was 3.15 (SD 0.22; range 1-4). The CVI average for the scale was 0.79 (SD 0.12). The overall average of face validity or clarity ratings was 3.44 (SD 0.25; range 1-4). The FVI average for the entire scale was 0.86 (SD 0.09). For all ratings, see Table 2. All items with scores below 0.78 for the FVI and CVI were further inspected, and adjustments were made to make the translated items clearer and more relevant. The full list of items of the original mARM and the translated mARM-G can be found in the Multimedia Appendix 1.

Cronbach α for the mARM-G was calculated first for the entire scale which was .91 (95% CI 0.88-0.94) and indicated excellent internal consistency. The value of Cronbach α for a whole scale, if an item is removed, remained highly consistent without any considerable difference (Table 3).

**Table 2:** Nonexperts (n=15) rating for content validity and face validity.

Items	Relevancy of each item, mean (SD)	I-CVI^a^	Clarity of each item, mean (SD)	I-FVI^b^
1	3.60 (0.51)	1	3.13 (1.13)	0.73
2	3.20 (0.94)	0.73	3.47 (0.92)	0.86
3	2.67 (0.82)	0.45	2.80 (0.86)	0.73
4	3.20 (0.94)	0.8	3.53 (1.06)	0.86
5	3.27 (0.96)	0.8	3.67 (0.62)	0.93
6	3.33 (0.72)	0.86	3.27 (0.80)	0.8
7	3.47 (0.64)	0.93	3.53 (0.64)	0.93
8	2.73 (1.03)	0.73	3.53 (0.92)	0.86
9	3.47 (0.92)	0.86	3.87 (0.35)	1
10	3.20 (0.86)	0.86	3.87 (0.35)	1
11	3.07 (0.96)	0.73	3.60 (0.83)	1
12	3.07 (1.10)	0.73	3.40 (0.63)	0.93
13	3.13 (0.64)	0.86	3.13 (0.99)	0.73
14	3.07 (0.96)	0.73	3.53 (0.74)	0.86
15	2.93 (1.03)	0.73	3.67 (0.62)	0.93
16	3.27 (0.88)	0.86	3.67 (0.49)	1
17	3.13 (0.83)	0.86	3.53 (0.74)	0.86
18	3.13 (0.74)	0.8	3.27 (0.88)	0.86
19	3.07 (0.80)	0.73	3.27 (0.96)	0.8
20	3.13 (0.99)	0.73	3.33 (0.98)	0.8
21	3.00 (0.93)	0.73	3.13 (0.99)	0.73
22	3.33 (0.82)	0.93	3.53 (0.64)	0.93
23	2.80 (0.77)	0.6	3.40 (0.63)	0.93
24	3.13 (0.83)	0.86	3.20 (1.01)	0.73
25	3.33 (0.82)	0.93	3.60 (0.51)	1
Measure average	3.15	0.79	3.44	0.86

^a^I-CVI: item–content validity index.

^b^I-FVI: item–face validity index.

**Table 3:** Reliability of mobile Agnew Relationship Measure German version if an item is removed.

Items	Cronbach α
1	.91
2	.90
3	.91
4	.91
5	.90
6	.90
7	.91
8	.90
9	.91
10	.90
11	.90
12	.91
13	.90
14	.90
15	.90
16	.91
17	.90
18	.90
19	.90
20	.91
21	.90
22	.90
23	.90
24	.90
25	.91

Cronbach α values were also computed for the specific subscales. These were taken from the description of the ARM [19] and were constructed of bond (items in the m-ARM-G: 2, 12, 14, 15, and 17; α=.76); confidence (items: 5, 6, 8, 11, 13, 16, and 19; α=.83); openness (items: 1, 4, 7, and 9; α=.61); partnership (items: 18, 21, 23, and 24; α=.77); and client initiative (items: 3, 10, 20, 22, and 25; α=.42) subscales. For the client initiative subscale, item 20 (“I am responsible for my recovery, not the app”) proved to be negatively correlated with the scale. The removal of this item from the mARM-G increased the α for the subscale to .55 (Table 4).

**Table 4:** Reliability of a client initiative subscale if an item is removed.

Items	Cronbach α
3	.39
10	.25
20	.55
22	.18
25	.31

**Figure 2:**
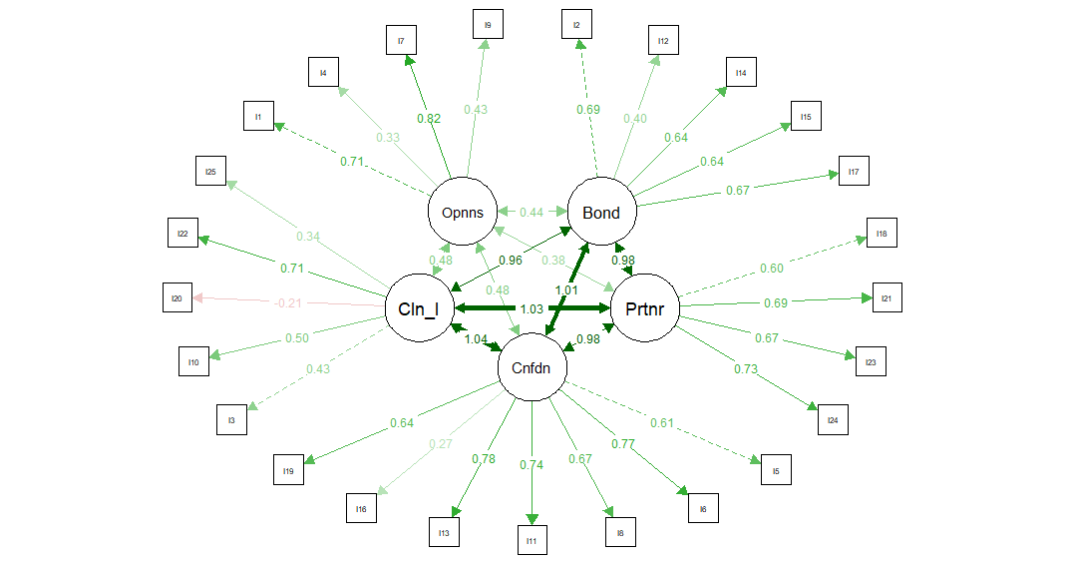
Visualization of confirmatory factor analysis results for the items loading onto the concepts openness (Opnns), bond (Bond), partnership (Prtnr), confidence (Cnfdn), and client initiative (Cln_I).

To further inspect the underlying structure of the translated measure, a confirmatory factor analysis was conducted. The chi-square statistic (*χ*^2^_265_=440.78, *P*<.001) was highly significant. The chi-square statistic to df ratio was 1.66. The Comparative Fit Index (CFI) was 0.87 and the Tucker-Lewis Index (TLI) was 0.86. The RMSEA was 0.07. The factor loading for item 20 was negative, and as such, it was removed from the measure (Figure 2).

## Discussion

### Principal Findings

TA refers to the working relationship between therapist and client [6] and has been found repeatedly to predict therapeutic outcomes [7-10]. Traditionally, TA has been widely assessed using the ARM [19]. With the digitalization of therapeutic practices, novel measures for TAs are required and there is an increased need to culturally adapt measures [21]. In this study, a German version of the mARM was developed and validated.

The results indicated high content validity with regard to relevance for the TA for the entire measure. Moreover, the translated items also proved to be easy to understand, with high ratings for the comprehensibility of the entire measure. Small adjustments were made upon suggestion, aiming to improve the comprehensibility of individual items. The study conducted reliability testing via Cronbach α. The entire scale had excellent internal consistency with α=.91. In addition, α values for the specific subscales were calculated as this is common practice for longer measures [36]. The subscales were taken from the original ARM [19]. We found good results for the bond, confidence, and partnership subscales (0.76-0.83), as well as close to acceptable results for the openness subscale (0.61). The client initiative subscale had poor results (0.42). These were further inspected, and item 20 (“I am responsible for my recovery, not the app”) was found to be negatively correlated with the overall scale and subscale. If removed, the α of the subscale increases to a value of .55, which equals the α results (.55) from the ARM [19]. The results are in line with the previous research using the ARM, which has reported ranges from good to acceptable results for the bond, openness, confidence, and partnership subscales; however, it has low internal consistency for the client initiative subscale [19,37].

To further inspect the underlying structure of the translated measure, a confirmatory factor analysis was conducted which revealed mixed results. The chi-square statistic (*χ*^2^_265_=440.78, *P*<.001) was highly significant and indicative of poor fit. The chi-square statistic to df ratio was 1.66, which indicates a good fit [38]. The CFI was 0.87 and the TLI was 0.86, which are below the suggested value of 0.90 [39]. The RMSEA was 0.07, which is acceptable [40]. The factor loading for item 20 was negative, and thus, the item was removed from the measure. We advise future researchers who use the mARM to evaluate the relationship between this item, the concept of client initiative, and TA overall with caution. Moreover, 6 items had factor loadings of <0.5 (items: 3, 4, 9, 12, 16, and 25). These items were further inspected; however, we ultimately decided against their removal since it would result in the openness and client initiative subscales being composed of only 2 remaining items.

### Limitations and Recommendations for Future Studies

This study had limitations. Reliability testing in this study was conducted with a group of students. Ratings for TA may thus be influenced by prior familiarity with mobile app use and overall technological familiarity. Moreover, this study used only 1 mobile app. Further research could therefore test the reliability of the measure with different apps, as well as using a sample of both younger and older participants with various socioeconomic backgrounds and various levels of experience with technology and mobile apps. This could further expand the literature on the generalization of TA measures in the broader population.

As there are still open questions concerning the nature and relation between digital TA and various treatment outcomes, future research could use the current scale to test associations between engagement and treatment outcomes, using both the overall scores of the mARM as well as the specific subscales. Further, obtaining more qualitative data concerning the nature of digital TA may yield novel insights.

Finally, additional studies comparing the effect of digital TA on treatment outcomes are needed for different therapeutic strategies, for example, for cognitive behavioral therapy, psychodynamic therapy, and acceptance-commitment therapy, among others, to gain a more nuanced understanding of the role of digital TA in various psychotherapy processes.

### Conclusions

With an increasing number of mHealth apps being developed and the need for research into the effectiveness and suitability of these novel innovations rising, the mARM-G presents an accurate, easily accessible, scalable, and low-cost measure to conduct further research in this novel area. This translation may prove to be of use since there are estimated to be more than 100 million native German speakers living across Germany, Austria, Switzerland, Belgium, Luxembourg, and Lichtenstein for whom this measure can be used [41].

The mARM-G and instructions are presented in Multimedia Appendix 2. We encourage researchers to use it freely for research and noncommercial evaluation of the TA between humans and mental health mobile apps.

## Appendix

Multimedia Appendix 1 Translation of the items from English to German.

Multimedia Appendix 2 German version of mobile Agnew Relationship Measure.

## Abbreviations

ARM: Agnew Relationship Measure
CFI: Comparative Fit Index
CVI: content validity index
FVI: face validity index
mARM: mobile Agnew Relationship Measure
mARM-G: mobile Agnew Relationship Measure German version
mHealth: mobile health
RMSEA: root mean square error of approximation
TA: therapeutic alliance
TLI: Tucker-Lewis Index

## Data Availability

The data sets generated and analyzed during this study are not publicly available due to the decision of the Ethics Committee of the Faculty of Arts and Social Sciences of the University of Zurich but are available from the corresponding author on reasonable request.
