# Supplementary material for: German Version of the Mobile Agnew Relationship Measure: Translation and Validation Study
Source: J Med Internet Res. 2023 Nov 13;25:e43368. doi: 10.2196/43368 (PMC10682917; doi:10.2196/43368)
Supplement: Multimedia Appendix 1 [file jmir_v25i1e43368_app1.docx]

| mARM English | mARM-G |
| --- | --- |
| I feel free to express the things that worry me | Ich kann mich zu den Dingen, die mich beschäftigen, frei äußern |
| I feel friendly towards the app | Ich habe eine positive Einstellung gegenüber der App |
| I take the lead when using the app | Ich übernehme die Führung, wenn ich die App nutze |
| I hold back some important things about myself from the app | Ich verschweige der App einige wichtige Dinge über mich |
| I have confidence in the app and the things it suggests | Ich habe Vertrauen in die App und ihre Vorschläge |
| I feel optimistic about my progress | Ich bin optimistisch in Bezug auf meinen Fortschritt |
| I feel I can openly express my thoughts and feelings when using the app | Ich empfinde, dass ich meine Gedanken und Gefühle offen ausdrücken kann, wenn ich die App nutze |
| I feel disappointed in the app | Ich bin von der App enttäuscht |
| I can share personal matters I am normally ashamed or afraid to reveal | Ich kann persönliche Dinge mitteilen, für die ich mich normalerweise schäme oder Angst habe, sie preiszugeben |
| I look to the app for solutions to my problems | Ich wende mich an die App, um Lösungen für meine Probleme zu finden |
| I have confidence in the app and how it works | Ich habe Vertrauen in die App und wie sie funktioniert |
| The app accepts me no matter how I respond | Die App akzeptiert mich, ganz gleich wie ich reagiere |
| The suggestions the app makes are important to me | Die Vorschläge der App sind wichtig für mich |
| The app seems to understand me | Die App scheint mich zu verstehen |
| The app’s feels warm and friendly with me | Ich fühle mich mit der App wohl und geborgen |
| The app does not give me the help I would like | Die App gibt mir nicht die Hilfe, die ich gerne hätte |
| The app is supportive | Die App ist unterstützend |
| The app seems to ignore my needs | Die App scheint meine Bedürfnisse zu ignorieren |
| The app confidently presents its information | Die App stellt ihre Informationen überzeugend dar |
| I am responsible for my recovery, not the app | Ich bin für meine Genesung verantwortlich, nicht die App |
| The more I use the app, the more I get out of it | Je mehr ich die App benutze, desto mehr habe ich von ihr |
| The app gives me the confidence to take the lead in my recovery | Die App gibt mir das Selbstvertrauen, die Führung in meiner Genesung zu übernehmen |
| I agree with the direction the app is taking me | Ich bin mit der Richtung, die die App einschlägt, einverstanden |
| The app is like having a member of my care team in my pocket | Mit der App ist es so, als hätte ich ein Mitglied meines Betreuungsteams in der Hosentasche |
| I am clear about what the app can and can't offer me | Ich bin mir darüber im Klaren, was die App mir bieten kann und was nicht |

*Changes made to the translated items:*

Item 4: Ich ergreife die Initiative, wenn ich die App benutze

Item 9: Ich kann der App persönliche Dinge mitteilen, für die ich mich normalerweise schäme oder Angst habe, sie preiszugeben

Item 16: Ich habe ein gutes Gefühl, wenn ich die App benutze

Item 24: Mit der App ist es so, als hätte ich einen Therapeuten stets bei mir in meiner Hosentasche
