# Supplementary material for: German Version of the Mobile Agnew Relationship Measure: Translation and Validation Study
Source: J Med Internet Res. 2023 Nov 13;25:e43368. doi: 10.2196/43368 (PMC10682917; doi:10.2196/43368)
Supplement: Multimedia Appendix 2 [file jmir_v25i1e43368_app2.docx]

|  | stimme überhaupt nicht zu | stimme eher nicht zu | stimme teils nicht zu | neutral | stimme teils zu | stimme eher zu | stimme voll und ganz zu |
| --- | --- | --- | --- | --- | --- | --- | --- |
| Ich kann mich zu den Dingen, die mich beschäftigen, frei äußern |  |  |  |  |  |  |  |
| Ich habe eine positive Einstellung gegenüber der App |  |  |  |  |  |  |  |
| Ich ergreife die Initiative, wenn ich die App benutze |  |  |  |  |  |  |  |
| Ich verschweige der App einige wichtige Dinge über mich |  |  |  |  |  |  |  |
| Ich habe Vertrauen in die App und ihre Vorschläge |  |  |  |  |  |  |  |
| Ich bin optimistisch in Bezug auf meinen Fortschritt |  |  |  |  |  |  |  |
| Ich empfinde, dass ich meine Gedanken und Gefühle offen ausdrücken kann, wenn ich die App nutze |  |  |  |  |  |  |  |
| Ich bin von der App enttäuscht |  |  |  |  |  |  |  |
| Ich kann der App persönliche Dinge mitteilen, für die ich mich normalerweise schäme oder Angst habe, sie preiszugeben |  |  |  |  |  |  |  |
| Ich wende mich an die App, um Lösungen für meine Probleme zu finden |  |  |  |  |  |  |  |
| Ich habe Vertrauen in die App und wie sie funktioniert |  |  |  |  |  |  |  |
| Die App akzeptiert mich, ganz gleich wie ich reagiere |  |  |  |  |  |  |  |
| Die Vorschläge der App sind wichtig für mich |  |  |  |  |  |  |  |
| Die App scheint mich zu verstehen |  |  |  |  |  |  |  |
| Ich habe ein gutes Gefühl, wenn ich die App benutze |  |  |  |  |  |  |  |
| Die App gibt mir nicht die Hilfe, die ich gerne hätte |  |  |  |  |  |  |  |
| Die App ist unterstützend |  |  |  |  |  |  |  |
| Die App scheint meine Bedürfnisse zu ignorieren |  |  |  |  |  |  |  |
| Die App stellt ihre Informationen überzeugend dar |  |  |  |  |  |  |  |
| Je mehr ich die App benutze, desto mehr habe ich von ihr |  |  |  |  |  |  |  |
| Die App gibt mir das Selbstvertrauen, die Führung in meiner Genesung zu übernehmen |  |  |  |  |  |  |  |
| Ich bin mit der Richtung, die die App einschlägt, einverstanden |  |  |  |  |  |  |  |
| Mit der App ist es so, als hätte ich einen Therapeuten stets bei mir in meiner Hosentasche. |  |  |  |  |  |  |  |
| Ich bin mir darüber im Klaren, was die App mir bieten kann und was nicht |  |  |  |  |  |  |  |

**Scoring**

The ratings from 1-7 for the 24 items are summed up, resulting in possible scores between 24 and 168. Items 4, 8, 16, and 18 are reverse coded.
